# NightLife: A cheap, robust, LED based light trap for collecting aquatic insects in remote areas

**DOI:** 10.3897/BDJ.4.e7648

**Published:** 2016-03-14

**Authors:** Benjamin Price, Ed Baker

**Affiliations:** ‡ Natural History Museum, London, United Kingdom

## Introduction

Inland waters cover less than 1% of our planet's surface, yet provide habitat to approximately one hundred thousand aquatic insect species, i.e. those with at least one aquatic lifestage (Balian et al. 2008, Dijkstra et al. 2014). Considering the taxonomic deficit in these groups this figure is likely a significant underestimate of the true aquatic insect diversity (Dijkstra et al. 2014).

The majority of aquatic insect diversity is comprised of true flies (Diptera), followed by caddisflies (Trichoptera), beetles (Coleoptera), dragonflies (Odonata), stoneflies (Plecoptera) and mayflies (Ephemeroptera) (Balian et al. 2008, Dijkstra et al. 2014). The ecology of these groups has been the focus of significant study due to their role as bioindicators of water quality, as many species are sensitive to pollution and sudden changes in their environment (Rosenberg and Resh 1993). In addition many aquatic dipteran species are vectors of disease (e.g. Currie and Adler 2008, Rueda 2008).

Aquatic insects are surveyed using a variety of methods including light trapping (e.g. Collier et al. 1997) which attracts emergent adults, and often mayfly subadults, using mercury vapour (MV) bulbs or actinic fluorescent tubes. Light trapping can be either active: attended light sheets, or passive: a combination of a light with a trap (Hardwick 1968, Hienton 1974). Passive traps allow samples from multiple sites to be collected in parallel by an individual in the field, with the number of sampling sites limited by the size and weight of each trap.

### Current Lights

Mercury vapour (MV) bulbs work by passing an arc of electric current through ionised mercury vapour; as a result these bulbs require a relatively high current to maintain the arc and thus are limited to use with either mains power or a petrol / diesel powered generator. Remote areas therefore cannot be sampled without significant effort. In addition MV bulbs are excessively bright for attracting aquatic insects, and tend to draw large numbers of night flying lepidoptera and other non-target species. MV bulbs are also delicate, easily damaged in transport and liable to break if exposed to rain during operation due to thermal fracture of the glass, and their high operating temperature is a waste of power.

Actinic fluorescent tubes also use mercury, but their method of operation requires a smaller current draw. While an improvement over MV bulbs, the current draw of fluorescent tubes does still require sizeable batteries if they are to be used in remote locations. For example a single 4W flourescent tube requires a 6v 12Ah battery weighing up to 2kg for an approximate 12hr run time. Fluorescent tubes are also delicate and liable to damage under field conditions.

Both MV bulbs and actinic fluorescent tubes contain mercury, which if released in the field can be hazardous for the environment.

An ideal aquatic insect light trap would have these properties:

1. Be able to run from small, standard, [potentially] rechargeable batteries.
2. Use low power light sources, at frequencies targeted for insect vision.
3. Be robust enough for field use without special packing or travel arrangements.
4. Be capable of autonomous operation.

### Light Emitting Diodes

Light Emitting Diodes (LEDs) are semiconductor devices that are used in a wide range of scientific, home and commercial lighting solutions due to the following properties:

- low power / high-efficiency (compared to incandescent / fluorescent)
- narrow spectral emissions (i.e. specific colours)
- long-life
- low-operating temperature
- durable (enclosed in a solid epoxy case rather than hollow glass)
- small size and weight

These same properties also lend themselves to the use of LEDs in insect collection.

### Insect Vision

Most insects studied have three types of photoreceptors: UV (λ_max_ ∼ 350 nm), blue (λ_max_ ∼ 440 nm) and green (λ_max_ ∼530 nm) (Briscoe and Chittka 2001). While some species are dichromats (UV + green) and others are sensitive to red light, the majority are sensitive to UV, blue and green light.

Many aquatic insects are nocturnal or crepuscular in the adult stage, and possess positive phototaxis: the attraction to light (Boda et al. 2014). In addition adult aquatic insects also possess positive polarotaxis: the attraction to horizontally polarized light, as a means of locating suitable aquatic habitats (Schwind 1991, Horváth 1995, Boda et al. 2014). The synergistic effect of phototaxis and polarotaxis can result in significant population reduction when lights interact with road surfaces near a body of water, resulting in females laying eggs on the road surface in error, especially with swarming aquatic species such as mayflies (Szaz et al. 2015).

### Existing use of LEDs in insect traps

Green LEDs have been used in a number of traps for catching horticultural pests including the Auchenorrhynchan virus vector *Bemisia tabaci* (Chu et al. 2003), the weevil *Euscepes postfasciatus* (Nakamoto and Kuba 2004) and various pests of commercially grown *Poinsettia* in glasshouses (Chen et al. 2004) as well as for species of veterinary importance including the mosquito genus *Culicoides* (Bishop et al. 2004).

The use of LED technology using light wavelengths suited for insect vision in insect traps has been studied previously by Cohnstaedt et al. (2008) using a patented system (Cohnstaedt et al. 2009). More recently Green et al. (2012) compared the efficancy of both white and UV LEDs to traditional actinic lights for sampling emergent aquatic insects using a modified Heath trap (Heath 1965); while there was no statistical difference between the actinic and UV LEDs the white LEDs resulted in a very reduced catch (Green et al. 2012).

However recent work by Pawson and Bader (2014) have shown that white LEDs are more efficient at attracting insects in general than yellow high-pressure sodium (HPS) lamps, and that the colour temperature of the white LED does not significantly influence the number of insects attracted, possibly due to the blue-green peak emmited by "white" LEDs, which is independent of their colour temperature.

In light of the previous work the aim of this study was therefore two-fold: (1) to build and field test a portable LED-based light-trap using LEDs targeting the UV, blue and green insect photoreceptors; and (2) to compare the targeted design against an actinic flourescent light (Philips Actinic BL TL 4W-10 UV-A G5) and a commercially available white LED bike light (Silva Simi white), given the potentially conflicting results of Green et al. (2012) and Pawson and Bader (2014).

## Materials and Methods

### Design and Fabrication

The NightLife traps are based on the Night Joule Thief circuit by Akimitsu Sadoi. The circuit comprises a modified joule thief circuit: a self-oscillating voltage converter designed to extract usable electrical energy from a cell, even when that cell is not capable of directly providing enough voltage to power the load circuit. The joule thief is used to power four wide angle 5mm LEDs (two UV, one blue, one green). The circuit is controlled automatically using a photoresistor, so that the LEDs will turn on without operator intervention in the field. The sensitivity can be adjusted using the potentiometer, allowing the trap to be set to come on at dusk, or full darkness.

**Figure 1.**
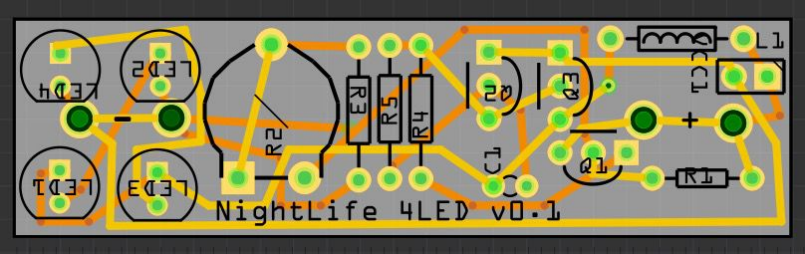
PCB design for Nightlife made using Fritzing. Copper traces complete the circuit between components. Traces run on the top surface (yellow) and bottom surface (orange) of the PCB. Text and figures in black are silk-screen printed onto the top surface.

**Figure 2.**
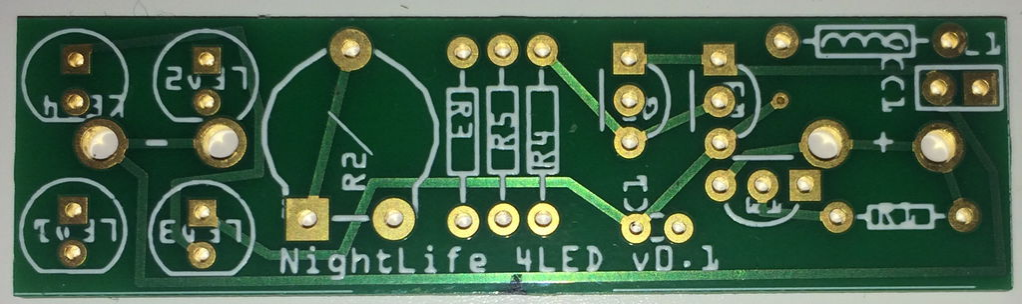
Commercially fabricated PCB from the design in Fig. 1

Design files for the Printed Circuit Board (PCB: Figs 1, 2) are available from the NHM Data Portal (Baker and Price 2015) in both the industry standard Gerber format, and for the open source Fritzing project. Fritzing (Knörig et al. 2009) allows small runs of boards (as few as one) to be ordered, although if a large number are needed, a traditional PCB fabrication company will offer significant cost savings.

**Figure 3.**
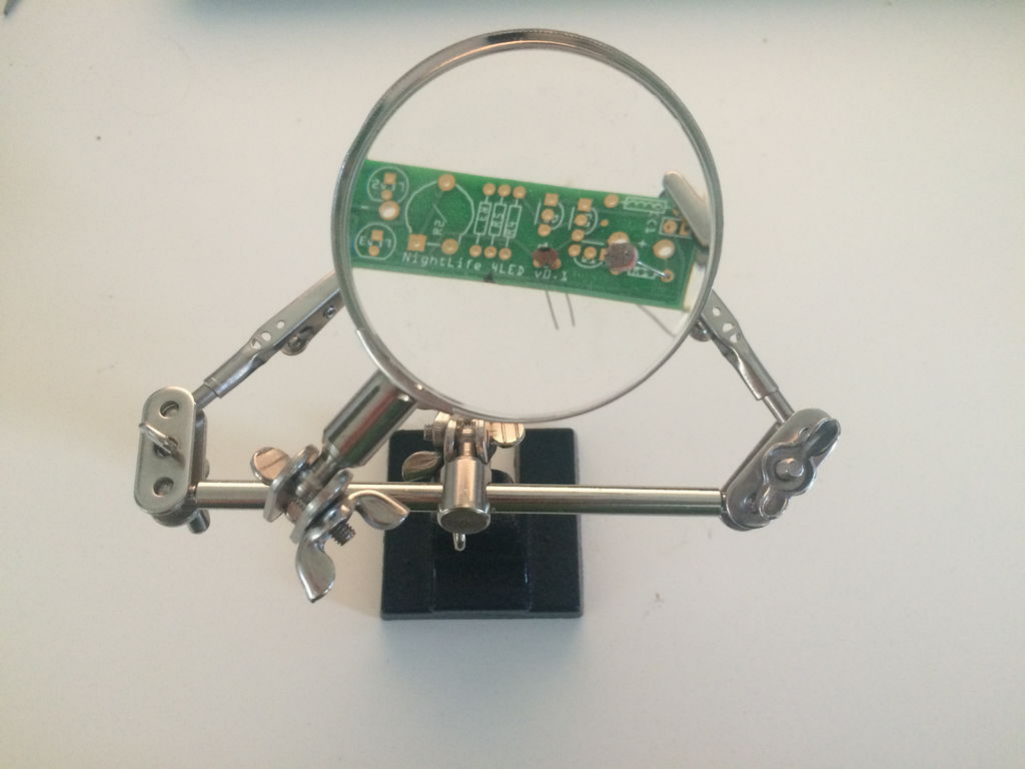
Assembly of a NightLife board using 'helping hands' tool for supporting the PCB.

Assembly of the device requires the following tools:

- Soldering iron (and solder)
- Wire cutters
- ‘Helping hands’ or other PCB holder (optional, Fig. 3)

**Figure 4.**
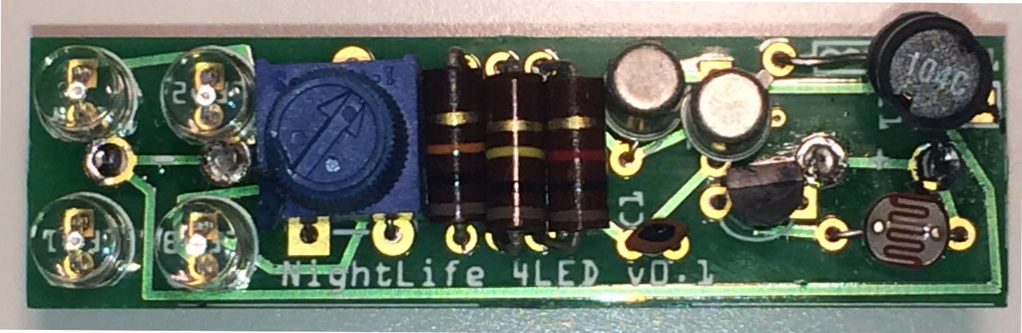
Completed NightLife board viewed from above. Total length = 50mm.

The transistors are the most thermally sensitive components and require care when soldering. The risk of damage can be minimised by ensuring the solder joint is made quickly, or by clamping the legs of the component (on the opposite side to the solder) with pliers to act as a heatsink. The other electronic components are thermally stable enough to survive relatively prolonged soldering. Fig. 4 shows a completed NightLife board.

**Parts List**

2 5mm UV LEDs [LED1; LED4]

1 5mm Green LED [LED3]

1 5mm Blue LED [LED2]

1 Photoresistor [R1]

1 Potentiometer [R2]

1 1KΩ resistor [R4]

1 10KΩ resistor [R3]

1 100KΩ resistor [R5]

1 22pF ceramic capacitor [C1]

1 470uH inductor [L1]

2 2N2222 NPN transistor [Q2; Q3]

1 2N2907 PNP transistor [Q1]

2 AA battery clips

These parts are best purchased in bulk (i.e. enough for 10 NightLife units) resulting in a total cost per unit of approximately £10 / €13 / $16 (currency conversion as at October 2014).

### Spectral Comparison

The spectral output of the 4W actinic fluorescent tube, the NightLife and the white LEDs were compared in laboratory settings using an Ocean Optics HR2000+ spectrometer and Ocean Optics SpectraSuite v1.6 (Ocean Optics Inc). Values were normalized for comparison between light sources by measuring from a standard distance and dividing by the integration time.

### Battery Life

Battery life was tested using by placing a NightLife device into a sealed carboard box while powered by a single fully charged Eneloop rechargable 2000mAh AA battery. The output of the light was measured using a photoresistor in a potential divider with a fixed resistor. The voltage across the photoresitor was recorded to a microSD card using an Arduino sketch over six discharges to obtain the mean duration of operation.

### Field Trials

The NightLife was trialed over a three night period in November 2014 on the Gudu and Mahai rivers of the Royal Natal National Park, South Africa (S: 28.6828; E: 28.9296). The Gudu river begins in the "Gudu Bush" an isolated patch of northern Afrotemperate forest (Mucina and Rutherford 2006) approximately 30 hectares in area and joins the Mahai river west of the Mahai rest camp.

Five sites were selected between the rest camp and the source of the Gudu river with the traps set during the day, and sensitivity set to maximum to turn the trap on as early in the evening as possible. The following day traps were recovered and either sorted to morphospecies (Ephemeroptera, Plecoptera and Trichoptera) or alternatively order level sorted (all other orders) with each sub-sample imaged in the field.

**Figure 5.**
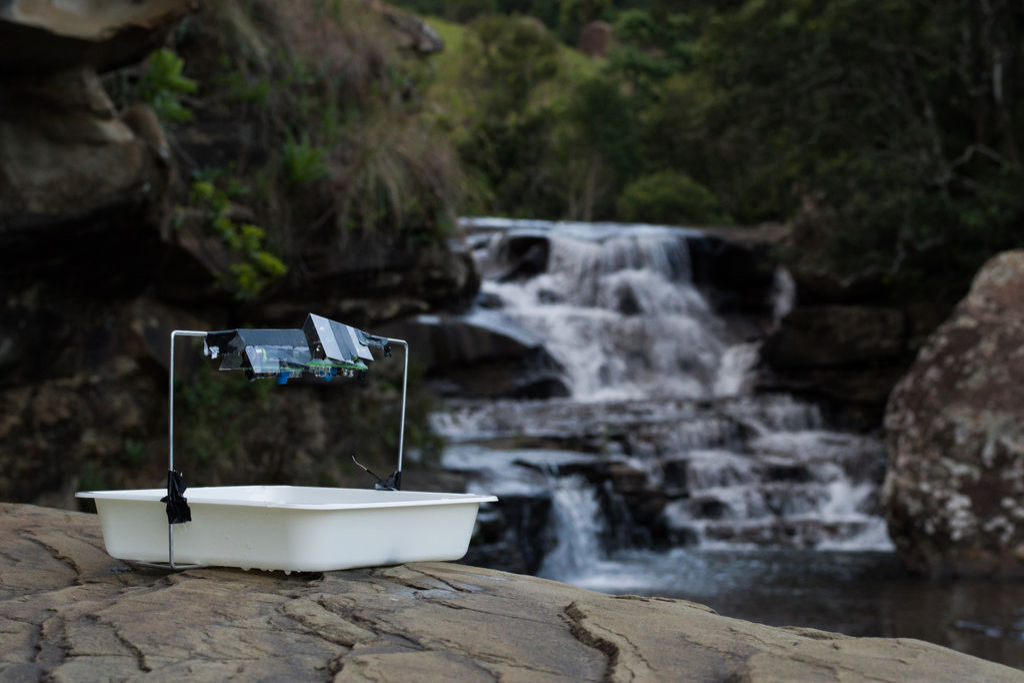
Two NightLife lights set up on a single pan trap and awaiting dusk before automatically turning on. Each device is protected by a clear rain shield.

**Figure 6.**
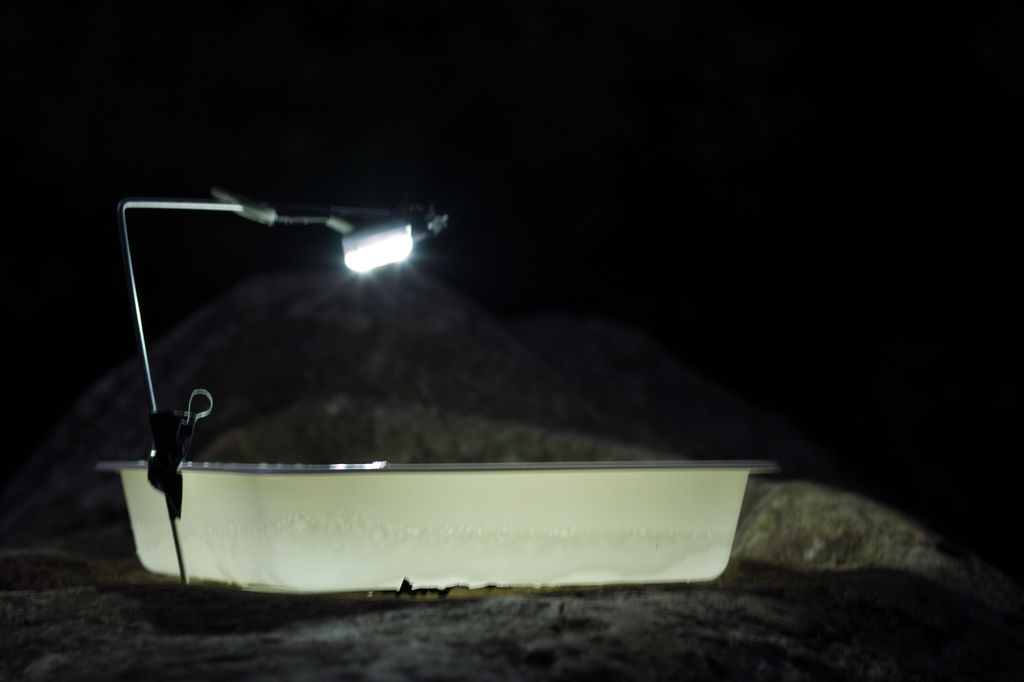
White LED bike light in operation.

Each trap consisted of either (A) two NightLife devices (eg. Fig. 5), (B) one white LED light (eg. Fig. 6) or (C) one 4W actinic tube, with the light source mounted 15cm over the centre of a white tray (25x25x5cm) pointing towards the tray. The tray was half filled with water and a drop of biodegradable liquid soap was added to break the surface tension. Two NightLife devices were used per trap due to the lower total light output of the NightLife in comparison to the other light sources when initially tested.

Due to limited materials and trapping nights on site the field trials focussed on testing the efficacy of the NightLife (6 traps, total of 12 trap nights) with limited comparison to the bike light (1 trap, total of 2 trap nights) and actinic tube (1 trap, total of 1 trap night). As a result comparisons are limited to qualitative (presence / absence) of particular orders and very basic comparisons of the Ephemeroptera, Plecoptera and Trichoptera morphospecies trap efficacy.

### Resin Casting

While the devices worked as expected in the field trials the exposed circuit of the NightLife PCB is vulnerable to oxidative damage when exposed to prolonged humidity or rainfall. In addition there is a risk of debris causing short circuits when the traps are in situ. The construction of enclosures that are both transparent and watertight is both complex and would dramatically increase the cost per unit. As a solution casting the NightLife board (apart from the battery clips) in clear polyester resin was trialled.

A mould was created by painting several layers of Latex over a block of Lego bricks of a suitable size. This mould was then peeled off of the Lego and filled with a well stirred mixture of polyester resin and a ‘catalyst’ (methyl ethyl ketone peroxide) to introduce free radicals to set the resin. The NightLife PCB is then suspended in the resin, to a level where all connections are immersed, but the battery clips remain accessible. Polyester resin is very clear when set, but takes a relatively long time (up to seven days in this case) to fully cure in a latex mould. To ascertain if the resin influenced the spectral output the enclosed version was compared to the exposed version used in the field trials as described previously.

## Data resources

Circuit board designs and the raw morphospecies collection data are available on the NHM Data Portal: http://dx.doi.org/10.5519/0060332

Analyses are available via GitHub: http://dx.doi.org/10.5281/zenodo.14048

## Results

### Battery Life

**Figure 7.**
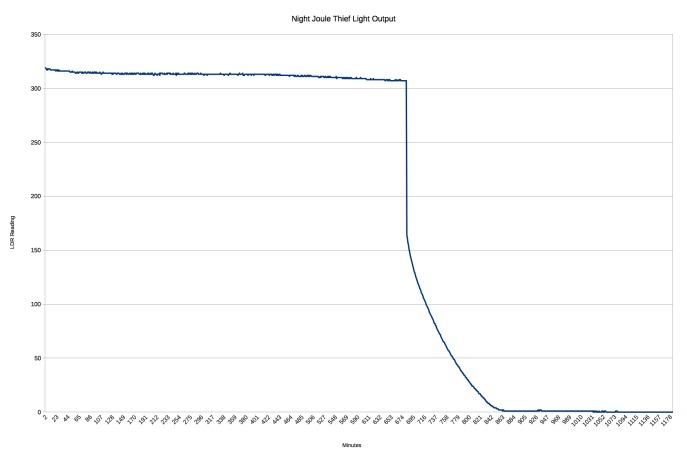
Typical light output (intensity) of a NightLife device during battery discharge.

A typical discharge curve is shown in Fig. 7. The mean time it took the measured output to halve was 660 minutes (11 hours), with output (>10% of initial) being detected for a mean of 804 minutes (13 hours). The characteristics of the joule thief circuit with the battery in a low charge state produce an oscillating voltage powerful enough to illuminate the LEDs. As the battery drains the rate of this oscillation decreases, eventually leading to a noticeable flickering effect. Mains and invertor driven bulbs also have oscillating output, fixed at the frequency of the supply voltage. The battery life of the white LED bike light was tested in the field, with a maximum recorded runtime of 16hrs on two Panasonic Lithium Coin Batteries (CR2032).

### Spectral Output

**Figure 8.**
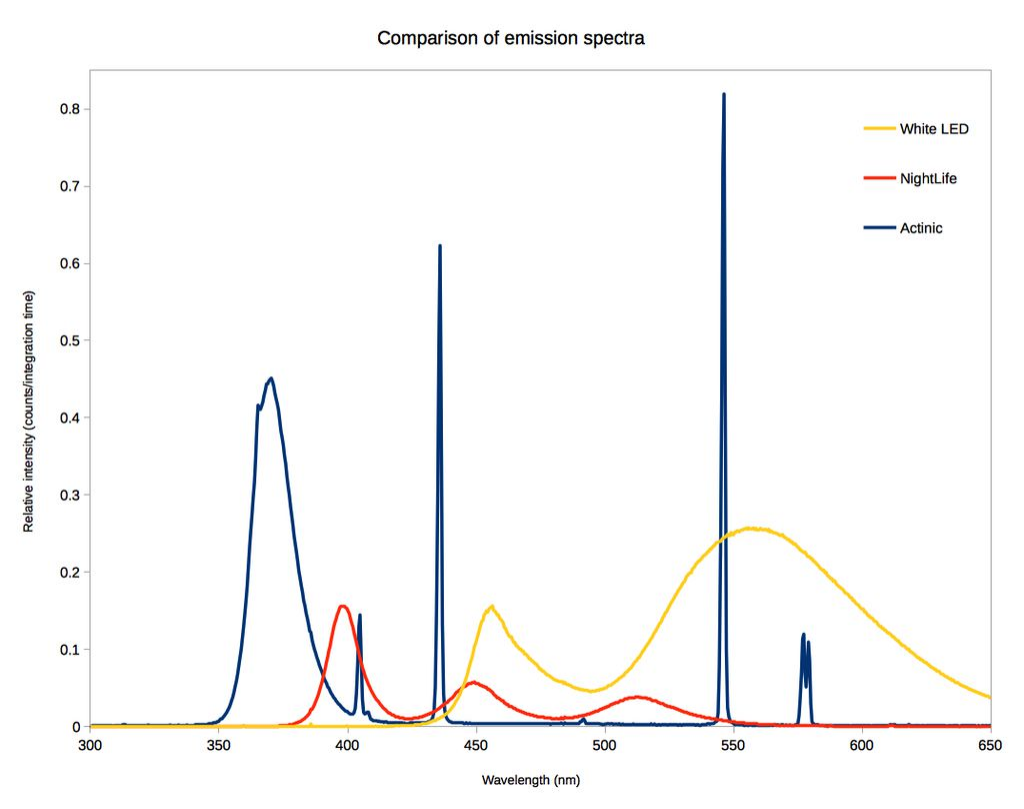
Spectra of 4W actinic fluorescent tube, NightLife and white LED light.

The output of NightLife is weaker than the actinic and white LED bulbs overall. The Nightlife output peaks at three distinct points: 397nm (long wave UV), 449nm (blue) and 512nm (green) (Fig. 8). However almost 50% of the light produced by the "UV" LED corresponds to violet light (400nm - 425nm). The actinic tube output peaks at five distinct points: 370nm (long wave UV), 404nm (violet), 436nm (violet), 546nm (green), and 577nm (yellow). The "white" LED bike light output peaks at two distict points: 456nm (blue) and 548nm (green).

### Field Trials

**Figure 9.**
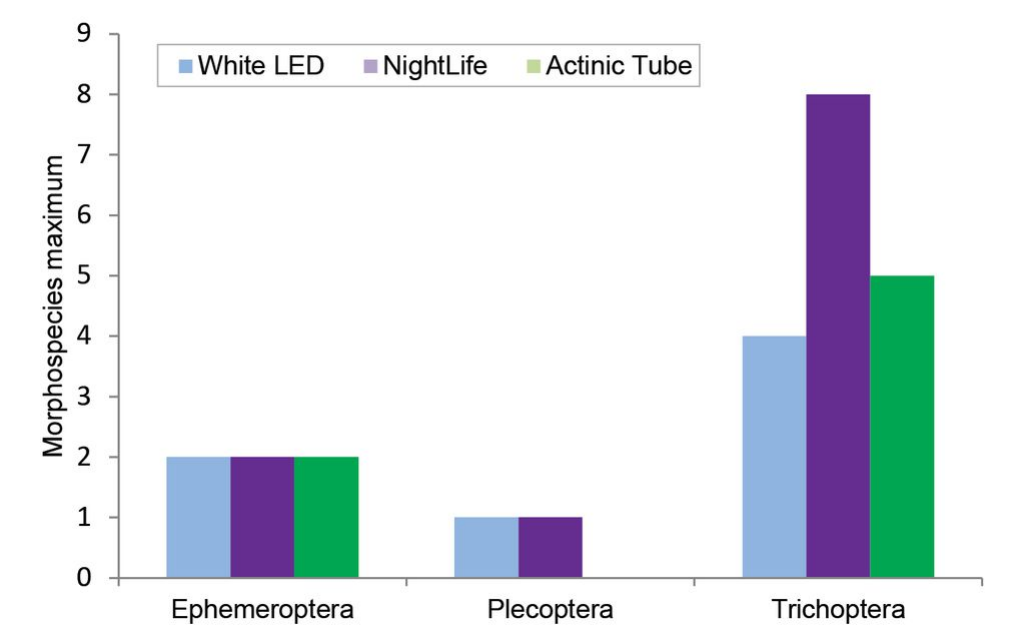
Comparison of the maximum number of Ephemeroptera, Plecoptera and Trichoptera morphospecies collected in a single trap during the field trial.

A total of 12 insect orders were collected by the NightLife, more than the white LED (9 orders) or the actinic trap (6 orders), however there was more opportunity to collect with the NightLife which likely skewed results (Table 1). All three trap types were dominated numerically by Diptera (Nematocera), followed by Lepidoptera and Trichoptera. The NightLife light collected more morphospecies of Trichoptera than the other two trap types (Fig. 9). The actinic trap did not collect any emergent Plecoptera, though this is almost certainly a result of the limited testing of this light source.

### Resin Casting

**Figure 10.**
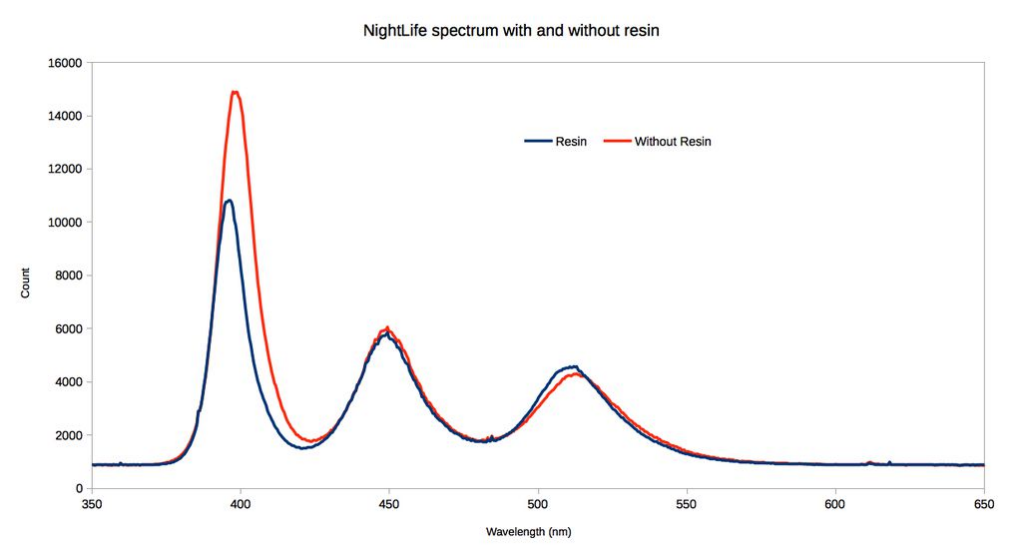
Comparison of emission spectrum of NightLife with and without being enclosed in polyester resin.

The emission spectra of two NightLife devices, one enclosed in resin and one as standard were determined. The resin has little effect on the (human) visible part of the spectrum, but does absorb approximately 30% of the UV light (Fig. 10​).

## Discussion

Light traps are an efficient way of sampling the emergent adults of freshwater habitats, however current lighting options are limited by the power requirements of actinic and MV bulbs. The NightLife LED based lightsource is a step towards the goal of a cheap, truly portable light trap for the emergent adults of freshwater insect species with a current cost of £10 per unit when produced in small runs.

### Light Output

**Composition.** The spectral composition of the NightLife is as expected, producing UV, blue and green light, although the UV LEDs emit light centred on the boundary between UV and violet light which is not optimal in comparison to the 350nm peak sensitivity of insect vision (Briscoe and Chittka 2001). The resin casting had little effect on the levels of blue and green light emitted, however the UV light was reduced by approximately 30%, which must be taken into consideration for future development. The actinic tube produces shorter wavelength UV, blue and green light which is more closely aligned with the peak sensitivity of insects to these wavelengths and explains the successful use of actinic bulbs in insect trapping. The white LED light produces a strong peak of blue light and a broad peak of green, yellow and red light, due to the phosphor coating which down-converts the blue light. As a result the white LED wastes much of its power producing yellow and red light which is of little use in attracting insects.

**Brightness.** The brightness of each light source, as estimated by the area under the curve (AUC) sensu Endler (1990) suggests that while the white LED is the brightest by a factor of six in the human visible spectrum (400nm - 700nm, AUC: white LED = 63; NightLife = 10; actinic = 8), this drops to a factor of two in the generalised insect visible spectrum (300nm - 600nm, AUC: white LED = 52, actinic = 28, NightLife = 13). This estimate of brightness does not take into account the specific sensitivity to certain wavelengths of light in insects (particularly UV) which may skew the results towards the actinic and NightLife lights in reality, as shown in the field trials. The low light output of the NightLife is to be expected, as the design of the device was focussed on low-power traps (using a single AA battery) and targeting specific wavelengths of light that are visible to insects, the success of which is demonstrated by the field trials.

Haitz's law, as described by Steele (2007), states that LED light output increases by a factor of 20 while cost per lumen decreases by a factor of 10 with each passing decade, being the LED equivalent of Moore's law (Moore 1965). Given that the current development of LED technology has surpassed this prediction it is almost certain that LED technology will be sufficiently powerful and efficient (as measured in lumens per watt) to be utilized in general insect light trapping in remote locations in the near future.

**Battery life.** The NightLife device has a comprable battery life to the actinic tube which equated to one night trapping at the Royal Natal National Park, with approximately 10 hours between sunset and the next sunrise at the time of field trials. The advantage of the NightLife device is that the light is autonomous and as a result multiple traps can be setup in advance throughout the day without subsequently wasting battery power before dusk. In addition a two AA batteries (per trap) are significantly lighter than the lead acid battery used by the actinic tube (61g vs 2kg).

### Trap Efficacy

**Figure 11.**
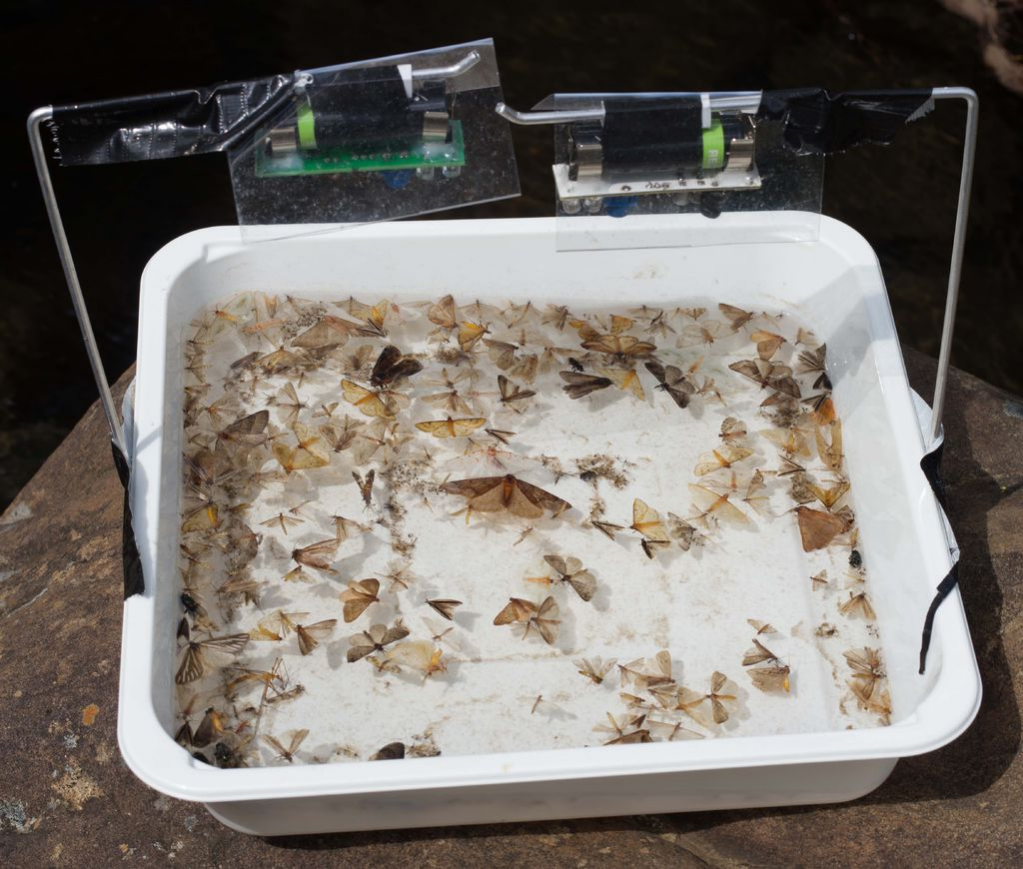
Specimens collected using the NightLife LED light, the traps were usually dominated by Diptera, Lepidoptera and Trichoptera.

The field trials show that the NightLife device is capable of attracting 12 orders of insects, including all of the aquatic insect orders typically collected by light trapping (Table 1) in high numbers. Comparison with the actinic light and white LED light was limited to qualitative measures given the reduced testing of the latter two systems in the field; however, we are confident that the NightLife device is capable of replacing the actinic setup in the field given the diversity and of insects collected with this device (Table 1, Figs 9, 11).

**Figure 12.**
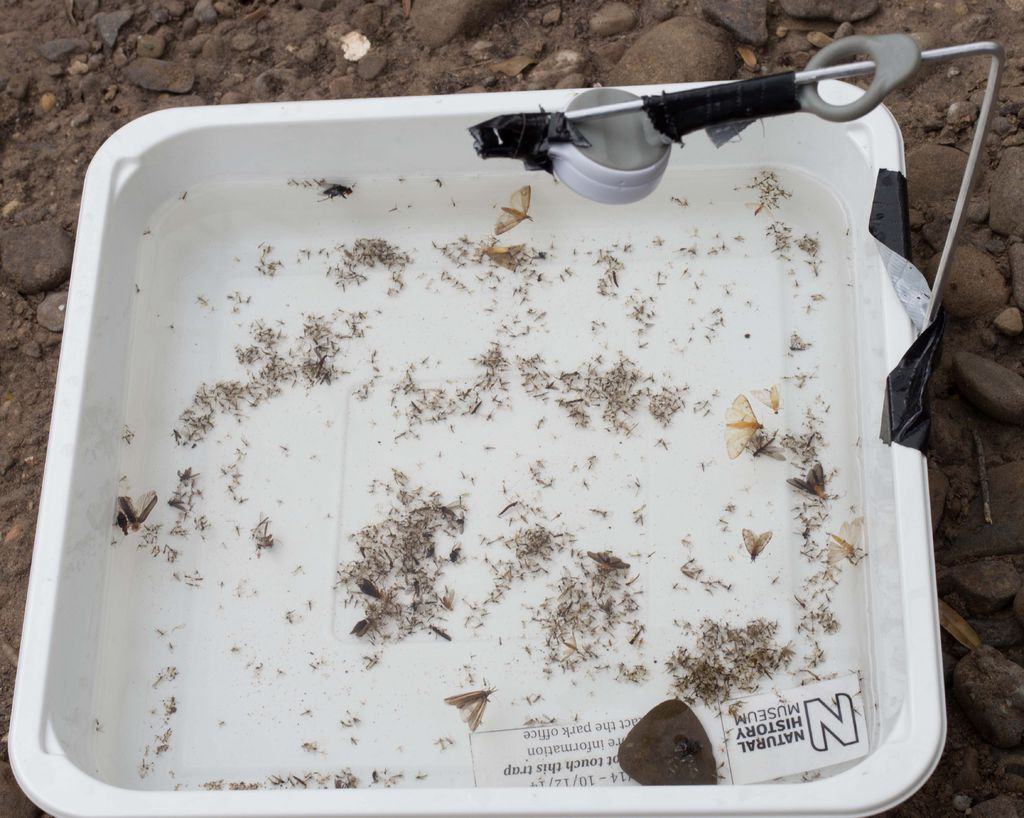
Specimens collected using the white LED light, note the low numbers of Lepidoptera in comparison to the NightLife trap.

The white LED light attracted representatives of all the aquatic orders usually collected, although there were fewer specimens attracted to the trap (in agreement with Green et al. 2012), especially Lepidoptera (usually by-catch) as the trap was dominated by Diptera (Fig. 12).

## Conclusions

Through developing NightLife we have demonstrated the feasibility of using small, lightweight and inexpensive LED based light traps for studying the biodiversity of aquatic insects.

### Applications

Studying the biodiversity of aquatic insects in remote areas, away from reliable sources of power, could be greatly aided by the use of the NightLife light source. Compared to other light sources the devices are small and robust, with similarly compact and reliable power sources (AA batteries). The low cost of the devices makes them a feasible alternative to more expensive solutions in developing countries, and could be used to greatly increase the number of sites sampled at once. The limiting factor on the number of sites sampled is no longer based on the cost and weight of the device, but on the number of sites which can be visited by the researcher(s) to setup and retrieve the traps.

### Potential for future development

**UV LEDs.** The UV LEDs trialled in the NightLife did not peak near the UV output of the actinic light (370nm) or the reported peak UV response of insect vision (350nm: Briscoe and Chittka 2001). Shorter wavelength UV LEDs are currently manufactured that emit either 370nm or 350nm. At present the 370nm UV 5mm LEDs cost approximately £2 each (in comparison the UV LEDs used were £0.20 each) and can be incorporated if required, however 350nm UV LEDs are currently prohibitively expensive. In future the manufacturing costs will likely reduce and these shorter wavelength UV LEDs will become a feasible addition to the NightLife trap without significantly increasing the cost.

**Solar power.** The integration of small solar cells into the design could be used to recharge the batteries during the day when the LEDs are turned off. This could drastically improve the unattended operational duration of the NightLife trap when combined with propylene glycol in the pan trap as a preservative.

**Additional data collection.** The augmentation of a light trap with low power sensors to measure abiotic variables, such as temperature and humidity (e.g. Baker 2014), could be used to increase the amount of ecological information from each trap.

## Figures and Tables

**Table 1.** Summary of insect orders collected using the three light sources, major aquatic groups are shown as bold. Only flying insects are reported, in addition the traps occasionally collected mites and spiders.

	**NightLife**	**White LED**	**Actinic**
** Ephemeroptera **	**×**	**×**	**×**
** Plecoptera **	**×**	**×**	
** Trichoptera **	**×**	**×**	**×**
** Diptera **	**×**	**×**	**×**
** Coleoptera **	**×**	**×**	**×**
Hemiptera	×	×	×
Hymenoptera	×	×	
Lepidoptera	×	×	×
Mantodea	×		
Mecoptera	×	×	
Neuroptera	×		
Psocoptera	×		

## References

[B2237298] Baker Ed (2014). Open source data logger for low-cost environmental monitoring. Biodiversity Data Journal.

[B2238111] Baker E., Price B. W (2015). Dataset: NightLife.. http://dx.doi.org/10.5519/0060332.

[B2400482] Balian E. V., Lévêque C., Segers H., Martens K. (2008). Freshwater Animal Diversity Assessment. Developments in Hydrobiology.

[B2236773] Bishop A. L., Worrall R. J., Spohr L. J., McKenzie H. J., Barchia I. M. (2004). Improving light-trap efficiency for Culicoides spp. with light-emitting diodes. Veterinaria Italiana.

[B2419387] Boda Pál, Horváth Gábor, Kriska György, Blahó Miklós, Csabai Zoltán (2014). Phototaxis and polarotaxis hand in hand: night dispersal flight of aquatic insects distracted synergistically by light intensity and reflection polarization. Naturwissenschaften.

[B2237255] Briscoe Adriana D., Chittka Lars (2001). The Evolution of Color Vision in Insects. Annual Review of Entomology.

[B2236787] Chen T-Y, Chu C-C, Henneberry T. J., K Umeda (2004). Monitoring and Trapping Insects on Poinsettia with Yellow Sticky Card Traps Equipped with Light-emitting Diodes. HortTechnology.

[B2236799] Chu C-C, Jackson C. G., Alexander P. J., Karut K., Henneberry T. J. (2003). Plastic Cup Traps Equipped with Light-Emitting Diodes for Monitoring Adult Bemisia
tabaci (Homoptera: Aleyrodidae). Journal of Economic Entomology.

[B2419418] Cohnstaedt Lee W., Gillen Jonathon I., Munstermann Leonard E. (2008). Light-Emitting Diode Technology Improves Insect Trapping. Journal of the American Mosquito Control Association.

[B2236823] Cohnstaedt L. W., Gillen J. I., Cohnsteadt W. M. (2009). Methods and compositions for improved light traps. US Patent.

[B2406054] Collier Kevin J., Smith Brian J., Baillie Brenda R. (1997). Summer light‐trap catches of adult Trichoptera in hill‐country catchments of contrasting land use, Waikato, New Zealand. New Zealand Journal of Marine and Freshwater Research.

[B2400719] Currie Douglas C., Adler Peter H. (2008). Global diversity of black flies (Diptera: Simuliidae) in freshwater. Hydrobiologia.

[B2400472] Dijkstra Klaas-Douwe B., Monaghan Michael T., Pauls Steffen U. (2014). Freshwater Biodiversity and Aquatic Insect Diversification. Annual Review of Entomology.

[B2480953] Endler John A. (1990). On the measurement and classification of colour in studies of animal colour patterns. Biological Journal of the Linnean Society.

[B2419428] Green Douglas, MacKay Duncan, Whalen Molly (2012). Next generation insect light traps: The use of LED light technology in sampling emerging aquatic macroinvertebrates. The Australian Entomologist.

[B2406705] Hardwick D. F. (1968). A brief review of the principles of light trap design with a description of an efficient trap for collecting noctuid moths. The Journal of the Lepidopterists' Society.

[B2419457] Heath J. (1965). A genuinely portable MV light trap. The Entomologists's Record and Journal of Variation.

[B2419255] Hienton T. E. (1974). Summary of investigations of Electric Insect Traps.

[B2419398] Horváth Gábor (1995). Reflection-polarization patterns at flat water surfaces and their relevance for insect polarization vision. Journal of Theoretical Biology.

[B2236853] Knörig André, Wettach Reto, Cohen Jonathan (2009). Fritzing: a tool for advancing electronic prototyping for designers.

[B2480943] Moore G. E. (1965). Cramming more components onto integrated circuits. Electronics Magazine.

[B2439857] Mucina L, Rutherford MC (2006). The Vegetation of South Africa, Lesotho and Swaziland. Strelitzia 19..

[B2480758] Nakamoto Yutaka, Kuba Hiroyuki (2004). The effectiveness of a green light emitting diode (LED) trap at capturing the West Indian sweet potato weevil, Euscepes
postfasciatus (Fairmaire) (Coleoptera: Curculionidae) in a sweet potato field. Applied Entomology and Zoology.

[B2236874] Pawson S. M., Bader M. F. (2014). LED lighting increases the ecological impact of light pollution irrespective of color temperature. Ecological Applications.

[B2400749] Rosenberg David M., Resh Vincent H. (1993). Freshwater Biomonitoring and Benthic Macroinvertebrates.

[B2400729] Rueda Leopoldo M. (2008). Global diversity of mosquitoes (Insecta: Diptera: Culicidae) in freshwater. Hydrobiologia.

[B2419408] Schwind Rudolf (1991). Polarization vision in water insects and insects living on a moist substrate. Journal of Comparative Physiology A.

[B2480933] Steele Robert V. (2007). The story of a new light source. Nature Photonics.

[B2419442] Szaz Denes, Horvath Gabor, Barta Andras, Robertson Bruce A., Farkas Alexandra, Egri Adam, Tarjanyi Nikolett, Racz Gergely, Kriska Gyorgy (2015). Lamp-Lit Bridges as Dual Light-Traps for the Night-Swarming Mayfly, Ephoron virgo: Interaction of Polarized and Unpolarized Light Pollution. PLOS ONE.

